# Swept Source Optical Coherence Tomography Assessment of Bursa Premacularis in Healthy Eyes

**DOI:** 10.1155/2020/7627128

**Published:** 2020-11-05

**Authors:** Paolo Carpineto, Rossella D'Aloisio, Daniele Guarini, Carla Iafigliola, Luca Cerino, Elisabetta Sciarroni, Luca Di Antonio, Katia Clemente, Marta Di Nicola, Giuseppe Di Martino, Rodolfo Mastropasqua, Lisa Toto

**Affiliations:** ^1^Ophthalmology Clinic, Department of Medicine and Science of Ageing, University “G. d'Annunzio” Chieti-Pescara, Chieti 66100, Italy; ^2^Department of Medical, Oral and Biotechnological Sciences, Laboratory of Biostatistics, University “G. d'Annunzio” Chieti-Pescara, Chieti 66100, Italy; ^3^Department of Medicine and Science of Ageing, School of Hygiene and Preventive Medicine, University “G. d'Annunzio” Chieti-Pescara, Chieti 66100, Italy; ^4^Institute of Ophthalmology, University of Modena and Reggio Emilia, Modena 41125, Italy

## Abstract

**Purpose:**

To describe the reliability and the limits of bursa premacularis (BPM) evaluation using a swept source optical coherence tomography (SS-OCT) device with enface and 16 mm-high definition (HD) longitudinal scans.

**Methods:**

60 eyes of 60 subjects were enrolled and imaged with SS-OCT system (PLEX Elite 9000, Carl Zeiss Meditec Inc., Dublin, CA, USA). BPM area was measured using enface scans imported to ImageJ. HD horizontal and vertical longitudinal scans centered at the fovea were used to detect width (W) and central thickness (CT) of BPM at baseline (T_0_) and after 30 minutes (T_30_) performed by two different observers. An enhanced vitreous visualization software provided by the manufacturer of the device was used to highlight vitreous structures.

**Results:**

BPM was identifiable in 100% of eyes using both horizontal and vertical longitudinal scans. On horizontal scan, BPM was not entirely measurable in 21.7% and in 18.3% of cases at T_0_ and T_30_, respectively. On vertical scan, BPM was not entirely measurable in 75.0% and in 81.7% at T_0_ and T_30_, respectively. No statistically significant differences were found between the two different time measurements with an intraclass correlation coefficient above 70%. Median BPM area was 26.9 (Q_1_-Q_3_: 19.5-40.5) mm^2^. In en face imaging, the most frequent BPM shape was the boat one.

**Conclusion:**

SS-OCT is a reliable tool for a detailed quantification and mapping of BPM, and it is able to add useful details about the morphological BPM features in youth population. However, the enhanced visualization of the vitreous structures is still a challenge, also with the most forefront devices.

## 1. Introduction

The Bursa Premacularis (BPM) represents a small reservoir of liquid vitreous located anteriorly to the fovea and is present in all eyes in the first decades of life [1–3].

Another distinct empty pocket of the posterior vitreous body and located over the optic disc is called the area of Martegiani (AM) [1–3].

Further observations were obtained in autopsy eyes in which the vitreous gel had been stained with fluorescein [4].

Later triamcinolone-assisted vitrectomy allowed intraoperative visualization of the BPM [5]. Time-domain optical coherence tomography (OCT) helped to depict the vitreous cortex when it was detached from the retina; however, the imaging failed to show the inner structure of the vitreous [6].

The advent of spectral-domain OCT (SD-OCT) technology has allowed better visualization of the vitreoretinal interface and posterior vitreous cortex through improved axial resolution, imaging speed, and signal-to-noise ratio.

Spectral-domain OCT was able to visualize the BPM in most eyes. However, because of the low sensitivity of the vitreous and limited scan length, SD-OCT often failed to measure the extension of the BPM [7].

More recently, swept source optical coherence tomography (SS-OCT) has been introduced in clinical practice for its axial depth and resolution and the capability to image the eye from vitreous to choroid.

Itakura et al. [8] first showed clarified boat-shaped BPM structure in vivo using single 12 mm nasal-temporal and 12 mm superior-inferior SS-OCT scans where brightness and contrast of the images were adjusted to enhance the vitreous.

Further studies [9–13] confirmed the utility of SS-OCT in defining and measuring the BPM.

The aim of our study was to verify the reliability and the limits of a recent commercially SS-OCT system to provide qualitative and quantitative in vivo mapping of BPM in healthy eyes of young subjects.

## 2. Material and Methods

### 2.1. Study Participants

In this observational cross-sectional study, 60 healthy young subjects (30 females, 30 males) were enrolled at the Ophthalmology Clinic of University “G. d'Annunzio” Chieti-Pescara, Italy. The study was approved by the local Institutional Review Board (IRB) (Department of Medicine and Science of Ageing, University “G. d'Annunzio” Chieti-Pescara) and adhered to the tenets of the Declaration of Helsinki. An IRB approved informed consent was obtained from all patients.

Inclusion criteria were (1) age between 18 and 35 eyes, (2) refractive error ≤ 6 D, (3) no history of previous ocular surgery included laser treatment, and (4) no systemic and ocular diseases.

Exclusion criteria were (1) axial length > 26 mm; (2) evidence of ocular conditions such as retinal detachment, retinal vascular occlusions, uveitis, and trauma; (3) evidence of systemic disorders, including diabetes and systemic hypertension; and (4) poor image quality or inadequate fixation.

One eye of each subject was chosen randomly if both eyes met the inclusion criteria. Randomisation was achieved using the random number generator Pro 2.17 (free software that is available on line).

All eyes enrolled were imaged with the SS-OCT PLEX Elite 9000 device (Carl Zeiss Meditec Inc., Dublin, CA, USA) between October 2019 and February 2020. Moreover, all patients received a complete ophthalmologic examination, which included the measurement of BCVA, intraocular pressure (IOP), ophthalmological evaluation, and biometry for axial length assessment (IOL Master 700, Carl Zeiss Meditec Inc., Dublin, CA, USA).

### 2.2. Image Acquisition

As gravity may affect the shape of the BPM, all subjects were invited to maintain their head straight for three minutes prior to begin both examinations.

Subjects underwent SS-OCT imaging using the PLEX Elite 9000 device (Carl Zeiss Meditec Inc., Dublin, CA, USA) which uses a swept laser source with a central wavelength of 1050 nm (1000–1100 nm full bandwidth) and operates at 100,000 A-scans per second.

FastTrac motion correction software was used, while the images were acquired.

Poor quality images (signal strength index (SSI) < 8) with either significant motion artifact or incorrect segmentation were excluded. En face scan was used to measure BPM area.

For each eye, 12 × 12 mm volume SS-OCT scans were acquired by two independent graders (ES and RDA), at baseline (T_0_) and after 30 minutes (T_30_). The following scans were considered: (1) HD Spotlight 1 (16 mm) (10-100x) a 0°, (2) HD Spotlight 1 (16 mm) (10-100x) a 90°, and (3) Cube (12 mm × 12 mm) (1024 × 1024 pixel).

### 2.3. Image Analysis

The main outcome measures were (i) width (W) and central thickness (CT) of BPM on longitudinal OCT scans at baseline (T_0_) and after 30 minutes (T_30_), (ii) BPM area on en face scans, and (iii) agreement between the two graders.

In detail, 16-millimeter horizontal and vertical longitudinal scans centered at the fovea were obtained to calculate W and CT of bursa using the device caliper function.

W and CT were detected on HD Spotlight horizontal and vertical scans, respectively.

**Figure 1:**
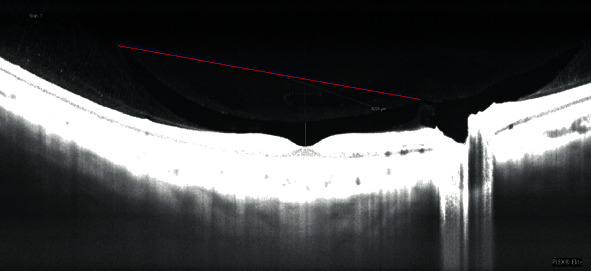
Width (W) and central thickness (CT) of bursa premacularis on longitudinal OCT scans. W was assessed considering a line combining the most distant points from foveal depression. CT was considered from foveal depression to superior border of bursa.

**Figure 2:**
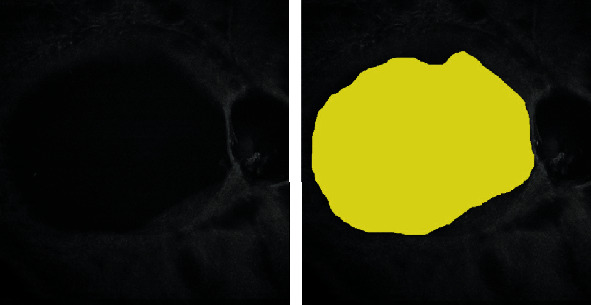
Bursa premacularis area assessment. For each eye, en face OCTA images were imported into the ImageJ software version 1.50, and consequently, BPM area was manually outlined in original scans.

W was assessed considering a line combining the most distant points from foveal depression (Figure 1). CT was considered from foveal depression to superior border of bursa (Figure 1). In order to enhance and then measure BPM area, we customized a 100-*μ*m thickness slab on coregistered b-scan of the en face images (Figure 2); briefly, the operator slightly moved the slab from inner limiting membrane to the vitreous cavity to highlight the hyperreflective roundish signal of the BPM, until its continuity was visualized.

For each eye, en face OCT images were imported into the ImageJ software version 1.50 (National Institutes of Health, Bethesda, MD; available at http://rsb.info.nih.gov/ij/index.html), and consequently, BPM area was manually outlined in original scans.

An enhanced vitreous visualization function of the device was used to highlight vitreous structures of all selected images.

**Figure 3:**
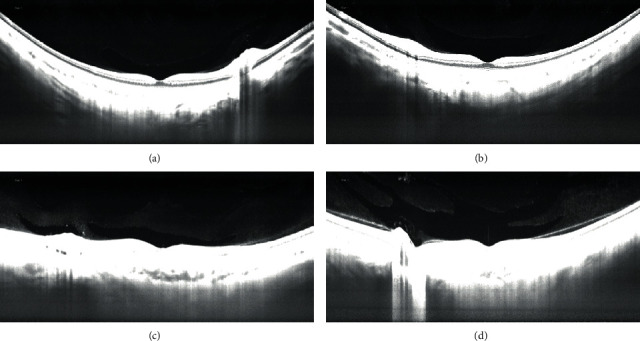
BPM morphology. BPM morphology was classified as a boomerang (a), as a boat (b), as a wedge (c), and as irregular (d).

The graders classified morphology of the bursa as boomerang, boat, wedge, or irregular shape using horizontal scans. In detail, BPM morphology was classified as a boomerang when the inferior and superior borders were wavy (Figure 3(a)). A boat shape was identified when the upper border was longer than the lower border (Figure 3(b)). A wedge shape was described when the lower border was longer than the upper border (Figure 3(c)). When neither classification was applicable, BPM was classified as irregular (Figure 3(d)).

### 2.4. Statistical Analysis

Qualitative variables were summarized as frequency and percentage; quantitative variables were summarized as median and interquartile range (expressed as Q_1_-Q_3_) according to their distribution. Departures from normal distribution were evaluated for each variable using a Shapiro-Wilk test. Intersession repeatability for each observer was measured using 2 measurements (T_0_ and T_30_). The intraobserver repeatability was evaluated by calculating the within-subject coefficient of variation (CVw) and intraclass correlation coefficient (ICC) with relative 95% confidence interval (95% CI). Bland-Altman plot was performed to assess the repeatability of the method by comparing repeated measurements for each single examiner, estimating the bias with the 95% confidence interval of the agreement. Lin's concordance correlation coefficient (CCC) with 95% CI was calculated to estimate the interobserver reproducibility. Spearman's Rho correlation coefficient was calculated to evaluate the correlation between axial length and BPM area. All tests were two-sided, and a level of statistical significance was set at *p* < 0.05. All the statistical analyses were performed using the R software environment for statistical computing and graphics version 3.5.2 (R Foundation for Statistical Computing, Vienna, Austria. https://www.R-project.org/).

## 3. Results

Demographic and clinical features of people enrolled (mean age 25.0 ± 3.1 years) are reported in Table 1.

Q_1_: first quartile; Q_3_: third quartile.

### 3.1. Horizontal and Vertical Longitudinal OCT Scanning Protocol

BPM was identifiable in 100% of eyes using both horizontal and vertical longitudinal scans.

On horizontal scan, BPM was not entirely measurable in 13 eyes (21.7%) and 11 eyes (18.3%) at T_0_ and T_30_, respectively, because it was hard to draw a clear demarcation line due to the bursa extension which was higher than the entire acquisition scan.

On vertical scan, BPM was not entirely measurable in 45 eyes (75.0%) and in 49 eyes (81.7%) at T_0_ and T_30_, respectively. W and CT measurements are reported in Table 2.

Q_1_: first quartile; Q_3_: third quartile.

The ICCs values reported in Table 3 indicate good reliability.

**Figure 4:**
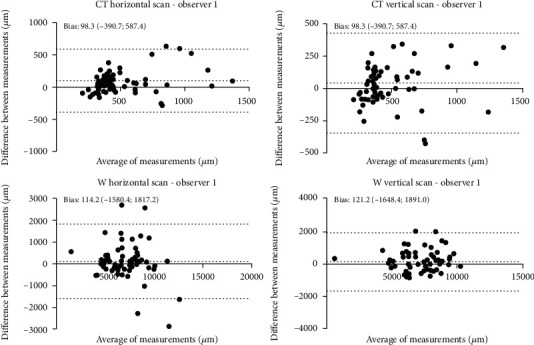
Bland-Altman concordance plot between T0 and T30 against their mean for observer 1. The lower and upper 95% limits of agreements are represented as dotted lines; the mean difference is represented as a continuous line. Statistical analysis was performed through Bland-Altman (*p* ≥ 0.05; paired *t*-test).

**Figure 5:**
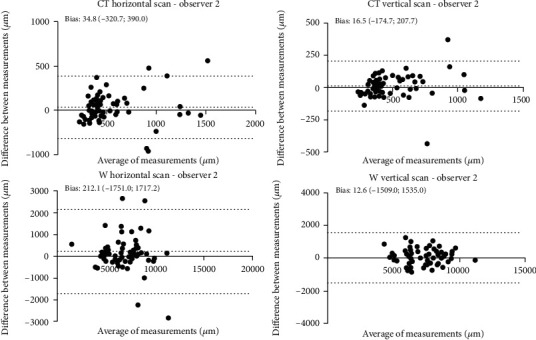
Bland-Altman concordance plot between T0 and T30 against their mean for observer 2. The lower and upper 95% limits of agreements are represented as dotted lines; the mean difference is represented as a continuous line. Statistical analysis was performed through Bland-Altman (*p* ≥ 0.05; paired *t*-test).

**Figure 6:**
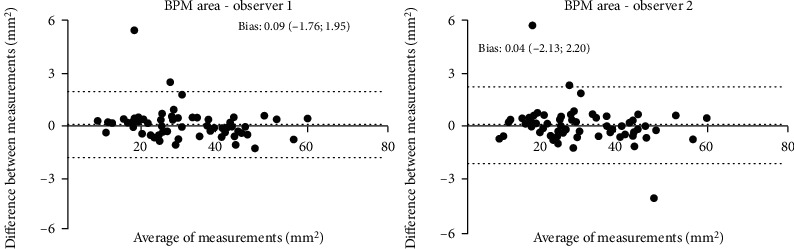
Bland-Altman concordance plot between T0 and T30 against their mean for observers 1 and 2. The lower and upper 95% limits of agreements are represented as dotted lines; the mean difference is represented as a continuous line. Statistical analysis was performed through Bland-Altman (*p* ≥ 0.05; paired *t*-test).

A Bland-Altman statistical analysis shows a good agreement between session providing good supporting evidence of the applicability of the SS-OCT device in quantifying BPM. The intersession agreements were plotted by the Bland-Altman plot, as reported in Figures 4–6.

Analysis using Lin's Concordance Correlation Coefficient confirmed a good agreement between measures obtained at T_0_ and T_30_ (CCC>0.8) for all parameters analyzed (Table 4).

### 3.2. En Face OCT Scanning Protocol

The median area of BPM was 26.9 (Q_1_-Q_3_:19.5-40.5) mm^2^ (Table 2).

In 5 eyes (8.3%), BPM size was not entirely measurable. BPM and AM were fused together in 36 eyes (60.0%). No significant correlation was found between bulbar axial length and BPM area (Rho = 0.160; *p* = 0.223). In our cohort of patients, boat shape was the predominant morphology (33/60 eyes, 55.0%). Wedge shape was observed in ten eyes (16.7%), boomerang shape in 6 eyes (16.7%), and irregular in 17 (28.3%).

## 4. Discussion

The liquid space in the posterior vitreous in front of the macula was first described as “bursa premacularis” by Worst [1] in 1976, based on preparations of postmortem vitreous. In 1987, Jongebloed and Worst, examining by coloured ink injection, the vitreous body of human eye removed postmortem, described connection between the BPM and other spaces in the vitreous. In 1990, Kishi and Shimizu [4] examined the vitreous of 84 human autopsy eyes by biomicroscopy after staining the gel component with fluorescein and immersing the specimen in water and found the presence of a posterior precortical vitreous pocket (PPVP) in all of 48 eyes with no or incomplete posterior vitreous detachment and in 19 of 36 eyes with posterior vitreous detachment. In addition, the posterior precortical vitreous pocket seemed to expand or become confluent with adjacent lacunae in the vitreous.

Later, Worst [14] described the existence of BPM anterior connections. However, also, Worst's observations were made in vitro on laboriously prepared postmortem specimens, possibly inducing further artifact.

In the last two decades, with the advent of sophisticated OCT devices, in vivo imaging of the posterior vitreous has been developed in order to better visualize its features and to report more details about mechanical properties of this biologic tissue.

Because of high penetration and high speed, SS-OCT was superior to SD-OCT for depicting the choroid and the vitreous compared, providing a longer range of vitreous imaging than previously used methods, thus allowing undisturbed visualization of the vitreous anatomy in vivo.

Using SS-OCT, Itakura et al. [8] examined the morphologic features of PPVP using swept source optical coherence tomography in normal subjects. In this study, SS-OCT was able to show the detailed structure of PPVP in vivo. The authors affirmed that the PPVP is probably the same space as the bursa premacularis described by Worst. The configuration of the PPVP was boat-shaped. Its central height, but not its width, increased with increasing myopia. In addition, a channel connected Cloquet's canal with the PPVP, so suggesting a possible route of aqueous humor into the PPVP.

An effective method to improve visualization of the vitreous using a technique based on SS-OCT was developed using dynamic focusing and windowed averaging.

Using this technology in 44 eyes of 25 subjects, who ranged in age from 23 to 62, Spaide demonstrated an optically empty space above the macula in all eyes, corresponding to the bursa premacularis. A conical space corresponding to the area of Martegiani was identified above the optic nerve head. The two hyporeflective areas were interconnected in 56.8% of cases. Anteriorly to the BPM, another hyporeflective space, named the supramacular bursa, was visible. It was separate from the BPM in horizontal scans centered on the fovea and was found in 38 eyes (86.4%). Both the supramacular and premacular bursae are interconnected anteriorly in 21 of the 38 eyes (55.3%) [9].

Many further information are not well known about these vitreous structures, firstly because of their transparency and secondly because of their continuous changes in shape with posturing, with ageing, and with myopic refraction [8]. The bursa premacularis tends to have a higher volume with ageing and height with myopic refraction [15] and may change shape and contour, even almost disappearing or simply move more anteriorly after a complete posterior vitreous detachment (PVD).

Vitreous changes are strongly related with ageing. Indeed, collagenous fibers of central vitreous body become thicker and more winding with ageing, with a posterior progressive migration of the vitreous cortex. This process is likely due to new collagen apposition anteriorly going towards preexisting vitreous and combining with it [16, 17].

An interesting recent study by Rossi et al. [12] used a Computational Fluid Dynamics method in order to evaluate the dynamics of the vitreous during saccadic motion and assess the impact of WSS on the retinal surface. It has been shown that eyeball could be exposed to exceptional mechanical stress due to the high angular velocity and acceleration of saccadic motion. The average WSS in the absence of the BPM was 4.5 times higher than eyes with BPM, suggesting a protective role of the bursa on the fovea. On the other hand, WSS showed a trend of increase in the periphery areas.

Our work aimed at analyzing quantitatively and qualitatively the BPM structure with an advanced and very recently commercially available device for a better comprehension of the vitreous morphology and topography.

To the best of our knowledge, the current study is the first research evaluating a forefront SS-OCT device with a wider field of scan as well as a longer wavelength and a faster scanning speed. Our results showed high reliability in repeated measurements of BPM in young population with an excellent agreement between the two operators. This aspect is essential considering that, as already known, BPM can change its position and shape very frequently, even with head posture.

Similarly to our study, a retrospective research analyzed posterior vitreous structures of 24 healthy individuals using a SS-OCT system with 12-millimeter horizontal and vertical longitudinal scans centered at the fovea. Conversely, we imaged 60 eyes using 16-millimeter HD longitudinal scans to better quantify the whole extension of BPM.

The latter was better explored with horizontal scan due to greater horizontal extension of this vitreal lacuna probably because of the stronger adhesion to the optic disc. Indeed, in our cohort, 60% of eyes showed the area of Martegiani fused with BPM. A progressive temporal extension of BPM has been previously observed during childhood growth. In addition, a previous work [18] has reported that BPM seems to move anteriorly with increased posterior vitreous detachment. The latter condition was not present in our cohort of healthy and young subjects suggesting a more posterior and horizontal position of BPM.

On the other hand, vertical scans showed more limitations in entirely quantifying BPM [18].

The most frequent shapes of such vitreal liquefied pocket are oval or round probably because of gravitational effects.

In our sample, we identified a new morphological pattern called boomerang shape, as well as the preexisting patterns already described by Park et al. [18] such as the boat, wedge, and irregular ones.

As expected, no significant correlation was found between AL and BPM size due to the low grade of myopia considered in our cohort of patients. It would be interesting to correlate BPM size and eyes with a longer bulbar axial length. Itakura et al. [8] described a central height increased with myopic refractive errors, but they did not considered en face imaging.

As known, anomalous PVD is associated with pathologic conditions, such as macular hole, vitreomacular traction syndrome, or epiretinal membrane [19].

Thus, by learning additional more qualitative and quantitative information about vitreous, we can expect further improvements in comprehension and management of vitreomacular pathologies.

Our results showed that BPM was identifiable in 100% of eyes using both horizontal and vertical longitudinal scans, confirming the high ability of SS-OCT to visualize vitreous structures. However, BPM was not completely detectable in 21,66% (T_0_) and 18,33% (T_30_) and in 75% (T_0_) and 81,66% (T_30_) on horizontal and vertical scan, respectively, suggesting a thorough quantification of the bursa is still challenging.

It would be advisable to implement OCT devices with a higher width and depth to entirely study the vitreous body and its lacune.

Our study has some limitations such as the restricted cohort of eyes with a medium myopia of the youth and the cross-sectional design. Further longitudinal studies are needed to investigate eyes with high myopia or vitreoretinal diseases as well. A better comprehension of vitreous anatomy may be a starting point to understand its changes with ageing and their correlation with the onset of vitreoretinal diseases. Furthermore, interesting would be to follow vitreous changes after vitreoretinal surgery to understand premacular bursa role in the development and progression of such vitreomacular pathologies.

## Figures and Tables

**Table 1:** Baseline characteristics of healthy subject enrolled.

Variables	
No of patients (eyes)	60 (60)
Age (yr), mean ± SD	25.0 ± 3.1
Gender (male/female)	30/30
Refractive error (diopters), median (Q_1_-Q_3_)	-0.9 (-2.0; -0.1)
Mean axial length (mm), median (Q_1_-Q_3_)	24.0 (23.3-24.3)

**Table 2:** Median and interquartile range (Q_1_-Q_3_) of scanned session at T_0_ and T_30_.

	Observer	T_0_	T_30_
CT horizontal scan (*μ*m)	1	462.5 (366.3-646.0)	402.0 (335.8-611.8)
	2	466.0 (366.5-654.5)	406.0 (337.5-615.2)
CT vertical scan (*μ*m)	1	419.5 (350.5-617.5)	382.0 (321.0-563.8)
	2	421.0 (352-621.2)	382.5 (322.2-566.5)
W horizontal scan (*μ*m)	1	7296.5 (5974.5-8501.8)	7067.5 (5937.0-8083.0)
	2	7247.0 (5978.5-8498.2)	7072.5 (5939-8082)
W vertical scan (*μ*m)	1	7372.0 (6082.3-8478.5)	7369.0 (5956.3-8261)
	2	7374.5 (6086.5-8480.5)	7372.5 (5957.75-8258)
BPM area (mm^2^)	1	26.9 (19.5-40.5)	26.6 (19.2-40.4)
	2	26.7 (19.4-39.1)	26.8 (19.2-40.4)

**Table 3:** Intrasession repeatability assessed by coefficient of variation (CVw) and intraclass correlation coefficient (ICC).

	Observer	CVw % (95% CI)	ICC (95% CI)
CT horizontal scan (*μ*m)	1	0.183 (0.150-0.220)	0.843 (0.750-0.903)
	2	0.184 (0.150-0.220)	0.843 (0.751-0.903)
CT vertical scan (*μ*m)	1	0.196 (0.160-0.230)	0.744 (0.605-0.839)
	2	0.197 (0.160-0.230)	0.743 (0.604-0.838)
W horizontal scan (*μ*m)	1	0.069 (0.030-0.110)	0.711 (0.597-0.822)
	2	0.070 (0.030-0.110)	0.708 (0.595-0.823)
W vertical scan (*μ*m)	1	0.062 (0.040-0.080)	0.870 (0.791-0.920)
	2	0.062 (0.040-0.080)	0.870 (0.791-0.920)
BPM area (mm^2^)	1	0.016 (0.009-0.024)	0.997 (0.995-0.998)
	2	0.019 (0.011-0.028)	0.996 (0.993-0.998)

**Table 4:** Interobserver reproducibility assessed by Lin's concordance correlation coefficient.

Observer 1 *vs.* observer 2	Lin's concordance correlation coefficient (95% CI)
CT horizontal scan (*μ*m)	
T_0_	0.855 (0.834-0.878)
T_30_	0.999 (0.997-0.999)
CT vertical scan (*μ*m)	
T_0_	0.999 (0.998-0.999)
T_30_	0.999 (0.997-0.999)
W horizontal scan (*μ*m)	
T_0_	0.848 (0.831-0.888)
T_30_	0.841 (0.828-0.890)
W vertical scan (*μ*m)	
T_0_	0.999 (0.996-0.999)
T_30_	0.999 (0.998-0.999)
BPM area (mm^2^)	
T_0_	0.999 (0.998-0.999)
T_30_	0.999 (0.997-0.999)

## Data Availability

The data used to support the findings of this study are available from the corresponding author upon request.
